# Supplementary material for: Time‑resolved multi-omic analysis of paclitaxel exposure in human iPSC‑derived sensory neurons unveils mechanisms of chemotherapy‑induced peripheral neuropathy
Source: Cell Death Dis. 2026 Feb 10;17(1):211. doi: 10.1038/s41419-026-08445-2 (PMC12921266; doi:10.1038/s41419-026-08445-2)
Supplement: Supplementary file 15 — Dataset 1. [file 41419_2026_8445_MOESM15_ESM.zip › CDD_Open_Data/04_RNA_Seq/Timeline_panel_plots_etc_2023-01-23_1609.zip!/Panel_Plots_etc/Evidence_Plots/post5d/evidence_plot_standard contrasts_msigdb_reactome_5d_PTX_vs_DMSO_ID6_M735_REACTOME_NEURONAL_SYSTEM.pdf]

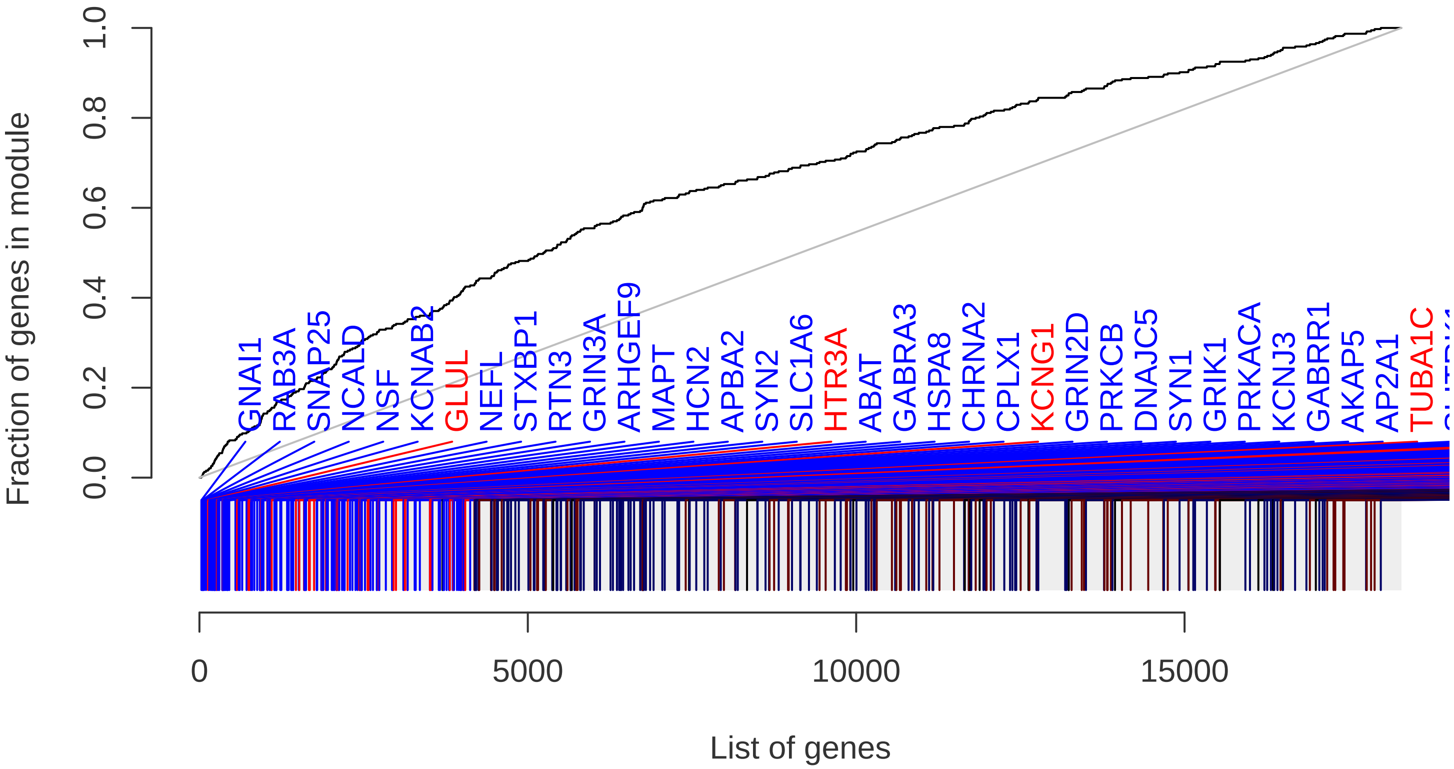
Fraction of genes in module
0.0
0.2
0.4
0.6
0.8
1.0
GNAI1
RAB3A
SNAP25
NCALD
NSF
KCNAB2
GLUL
NEFL
STXBP1
RTN3
GRIN3A
ARHGEF9
MAPT
HCN2
APBA2
SYN2
SLC1A6
HTR3A
ABAT
GABRA3
HSPA8
CHRNA2
CPLX1
KCNG1
GRIN2D
PRKCB
DNAJC5
SYN1
GRIK1
PRKACA
KCNJ3
GABRR1
AKAP5
AP2A1
TUBA1C
0
5000
10000
15000
List of genes
